# Importance of brucellosis control programs of livestock on the improvement of one health

**DOI:** 10.1080/01652176.2021.1894501

**Published:** 2021-03-08

**Authors:** Maryam Dadar, Ruchi Tiwari, Khan Sharun, Kuldeep Dhama

**Affiliations:** a Razi Vaccine and Serum Research Institute, Agricultural Research, Education and Extension Organization (AREEO), Karaj, Iran.; b Department of Veterinary Microbiology and Immunology, College of Veterinary Sciences, UP Pandit Deen Dayal Upadyaya Pashu Chikitsa Vigyan Vishwavidyalya Evam Go-Anusandhan Sansthan (DUVASU), Mathura, Uttar Pradesh, India; c Division of Surgery, ICAR-Indian Veterinary Research Institute, Bareilly, Uttar Pradesh, India; d Division of Pathology, ICAR-Indian Veterinary Research Institute, Bareilly, Uttar Pradesh, India

**Keywords:** Bovine, cattle, brucellosis, livestock production, control programs, milk, safety

## Abstract

Brucellosis not only represents an important health restraint on livestock but also causes high economic losses in many developing countries worldwide. Despite considerable efforts made for the control of brucellosis, the disease is still spreading in many regions (such as the Middle East) where it represents one of the most important health hazards impacting both animals and humans. The present review aims to investigate the efficacy of veterinary control programs regarding brucellosis, with a special focus on current prevention, control, and eradication approaches. The reasons for unsuccessful control programs such as the absence of highly effective vaccines and non-certified bulls are also debated, to understand why the prevalence of brucellosis in livestock is not decreasing in many areas despite considerable efforts taken to date. The importance of governmental and regional investment in brucellosis control remains one of the main limiting factors owing to the limited budget allocated to tackle this disease. In this context, one health concept has generated novel comprehensive approaches with multiple economic implications across the livestock industry and public health. However, the implementation of such global preventive strategies appears to be a key issue for many endemic and low-income countries. According to the collected data, epidemiological contexts including management and trade systems along with well-defined agro-ecological zones should be evaluated in brucellosis endemic countries to improve milk production and to enhance the sustainability of the livestock sector at both national and regional levels.

## 1. Introduction

Brucellosis is a contagious and widespread zoonotic disease of domestic and wild animals in different regions worldwide (Franc et al. 2018). *Brucella* spp. are non-spore-forming pathogens, non-motile aerobic Gram-negative coccobacilli with small size of 0.6–1.5 μm in length and 0.5–0.7 μm in diameter that categorized in the family of Brucellaceae. The family Brucellaceae comprises the genus *Brucella* and six further genera, including *Ochrobactrum*, *Daeguia*, *Crabtreella*, *Mycoplana*, *Pseudochrobactrum*, and *Paenochrobactrum* which are phylogenetically members of the order Rhizobiales within the class Alphaproteobacteria (Leclercq et al. 2020). Twelve species are currently described in the genus of *Brucella* that infect different wildlife and domestic animal species (Whatmore et al. 2016). Among these, six *Brucella* species have been categorized according to their pathogenicity and preferred hosts as *Brucella abortus* (cattle), *B. melitensis* (goats and sheep), *B. ovis* (rams), *B. canis* (dogs), *B. suis* (pigs), and *B. neotomae* (Common voles, desert wood rat). The most important pathogenic species in man are known as *B. melitensis, B. suis,* and *B. abortus* (Omer et al. 2000; Lindahl et al. 2014; Wareth et al. 2014; Kaynak-Onurdag et al. 2016; Whatmore et al. 2016). Recently, two new *Brucella* spp., *B. ceti* (dolphins, porpoises and whales), and *B. pinnipedialis* (walruses and seals) have been reported from marine mammal hosts according to their pathogenicity and preferred hosts (Cvetnić et al. 2016; Ness et al. 2017). Furthermore, *Brucella* species such as *B. inopinata* have been isolated from humans (Scholz et al. 2010; Olsen and Palmer 2014) and *B. papionis* sp. nov. along with new *Brucella*-like coccoid bacteria also were reported from baboons (Papio spp.) (Whatmore et al. 2014). Apart from well-known endemic regions, brucellosis remains a neglected disease in many areas worldwide which can lead to serious health and economic concern for the livestock populations by affecting animals such as cattle, buffalo, camel, sheep and goat (Sulima and Venkataraman 2010; Santos et al. 2013; Mableson et al. 2014; Bamaiyi 2015; Singh et al. 2015). Economic losses of brucellosis for the livestock industry and small-scale livestock holders are usually due to infertility in both sexes and late term abortion (Sulima and Venkataraman 2010; Angara et al. 2016; Awah-Ndukum et al. 2018; Deka et al. 2018; Franc et al. 2018), decreased milk yield (Herrera et al. 2008; Mellado et al. 2014), decreased productivity, loss in market value of animals, lost draught power, missed reproductive cycles, birth of weak offspring with low birth weight, and increased veterinary costs in farms (Blasco and Molina-Flores 2011; Dadar et al. 2020a). In the cows, the disease leads to an abortion once in their lifetime and the infection is asymptomatic in non-pregnant animals which may remain infected their entire life (Godfroid et al. 2010). Moreover, it has been reported that the annual economic losses and prevalence of brucellosis are variable in different countries and *Brucella* vaccines alone could not eradicate brucellosis, particularly in regions showing high levels of *Brucella* infection (Seleem et al. 2010; Awah-Ndukum et al. 2018).

Therefore, vaccination combined with proper measures of husbandry is more likely to achieve successful prevention and control of *Brucella* infections. However, the complexity of brucellosis control in different endemic countries may offset the preventive effects of applicable long-term intervention approaches on livestock, thereby causing significant economic losses for veterinary instances (Singh et al. 2015; Deka et al. 2018). Such losses are not confined to the livestock production (reduced milk, delayed conception and abortion) (Bamaiyi 2015; Avila-Granados et al. 2019; Machavarapu et al. 2019), rather extending over the global public health system (cost of treatment and productivity loss) (Dadar et al. 2019b).

This review focuses on the importance of veterinary control programs regarding brucellosis, with a special focus on the current prevention, control, and eradication approaches.

## 2. Pathogenesis of *Brucella* spp. and involved immune mechanisms

*Brucella* spp. could proliferate within the macrophages and escape from host defense mechanisms and can infect human host by contact with mucosa or inhalation, puncture wounds such as needle sticks as well as ingestion (Hull and Schumaker 2018). *Brucella* needs four steps to infect the host, namely containing adherence, invasion, establishment, and dissemination within the host (Christopher et al. 2010). The pathogen can survive in the macrophages and then multiply and control the fusion of phagosome–lysososme complex (Gopalakrishnan et al. 2016). After that, the accumulated bacteria are circulated to other cells of host (Ko and Splitter 2003). Moreover, it has been reported that there are five virulence factors for *Brucella* spp. which are necessary for infection and intracellular survival, including cyclic β-glucan (Martirosyan et al. 2012), virB T4SS (de Jong et al. 2013), pathogen-associated molecular patterns (PAMPs), two component sensory and regulatory system BvrS/BvrR (Martín-Martín et al. 2012), and *Brucella* LPS (BrLPS) (Lapaque et al. 2005). Furthermore, other virulence factors in *Brucella* spp. are comprised of outer membrane proteins (Omps) (Lim et al. 2012; Vizcaíno and Cloeckaert 2012), BacA (Martín-Martín et al. 2012), SagA (Del Giudice et al. 2013), BmaC (Posadas et al. 2012), BetB (Lee et al. 2014), BtaE (Ruiz-Ranwez et al. 2013) and MucR (Mirabella et al. 2013). Also, a genomic island (GI_FeGSH_) is associated with pathogenicity of *Brucella* which has been described in the genome of *B. canis* (Wahab et al. 2017). However, this bacterium does not contain any plasmid associated with the pathogenicity of its genome that make it different from other bacterial species (Bano and Lone 2015). Moreover, the genome also lacks the numerous other common virulence genes such as fimbriae, antigenic variation, capsules, cytolysins, exotoxins, resistance forms, plasmids, or lysogenic phages (Detilleux et al. 1990; Moreno et al. 2002). Several antigenic components of *Brucella* such as Omp16, Omp19, Omp25, Omp31, SurA, Dnak, trigger factor (TF), ribosomal protein L7L12, bacterioferritin (BFR) P39, and lumazine synthase BLS have been characterized (Bundle et al. 1989; Barrionuevo and Giambartolomei 2019; Yin et al. 2019). Efforts were instead concentrated on surface antigens (described as R in rough strain and A, M in smooth strains) and lipopolysaccharide (LPS) expected to trigger specific antibody response (Mancilla 2016; Głowacka et al. 2018; Jezi et al. 2019). The specific structure of LPS in the *Brucella* confers resistance to antimicrobial drugs, while inducing a very low endotoxicity. The LPS of *Brucella* also organizes the virulence factor and regulates the intracellular replication as well as its survival in the host (Christopher et al. 2010; Atluri et al. 2011). LPS comprised of lipid A, O-antigen and oligosaccharide core in Gram-negative bacteria (Głowacka et al. 2018). It has also been reported that antigen M predominates in *B. melitensis*, whereas antigen A is predominant in *B. suis* and *B. abortus* (Casabuono et al. 2017; Kumar et al. 2019). Moreover, lipid A containing unique fatty acids, excepting ß-hydroxymyristic acid and two types of aminoglycose, are the main components in the LPS of smooth phase strains (S-LPS). The core region of *Brucella* S-LPS is composed of glucose, mannose, and quinovosamine with an O chain that contains a homopolymer of nearly 100 residues of 4-formamido-4,6-dideoxymannose (Fontana et al. 2016; Smith 2018). Several studies using murine models revealed that the main host response to *Brucella* spp. could be attributed to T helper 1 (Th1), along with production of gamma interferon (IFN-γ) by natural killer (NK) cells and T cells (Copin et al. 2007; Rolán and Tsolis 2008). Moreover, both CD4 and CD8 T cells improve the limitation of *Brucella* infection, that may reveal their function as IFN-γ sources (Araya et al. 1989). Furthermore, *B. abortus* could stimulate the release of anti-inflammatory cytokine interleukin-10 (IL-10) along with an early Th_1_ response (Svetić et al. 1993; Xavier et al. 2013). However, the macrophages, B cells, and CD4 T cells could produce the IL-10 which can restrict the antagonizing role of IFN-γ, as well as the microbicidal activity of macrophages against *Brucella* (Fernandes and Baldwin 1995).

## 3. Economic losses due to brucellosis

Economic losses associated with brucellosis in livestock have been reported in different countries (Islam et al. 1983; McDermott et al. 2013; Santos et al. 2013; Singh et al. 2015). Although estimates of the costs associated with brucellosis infections remain limited to specific countries, all data suggest that worldwide economic losses due to brucellosis are extensive regarding both livestock health, production and public health (cost of treatment and productivity loss in man) (Sriranganathan et al. 2009). For example, epidemiological surveys conducted in India reported a median US $3.4 billion estimated economic loss due to livestock brucellosis (Mantur and Amarnath 2008; Singh et al. 2015, 2018; Machavarapu et al. 2019). In another official report, the annual economical losses due to bovine brucellosis have been reported at approximately $600 million in Latin America (Sriranganathan et al. 2009). A 20–30% decrease in milk production has been estimated in brucellosis-affected farms (Herrera et al. 2008; Havelaar et al. 2019). A few countries provide accurate reports regarding their losses due to brucellosis such as Argentina, with an annual loss reaching US $60 million (Samartino 2002), India with a median loss of US $3.4 billion (Singh et al. 2015) for cattle, sheep and goat, Egypt with US $9.8 million (Bamaiyi 2015), United States with US $30 million (Bittner 2004), Brazil with approximately US $448 million (Santos et al. 2013), and Kyrgyzstan about US $10.6 million (Bamaiyi 2015). In Nigeria, the annual economic losses caused by brucellosis in small ruminants were US $3.2 million two decades ago (Brisibe et al. 1996). However, the brucellosis eradication programs may be very expensive in developing countries (Zhang et al. 2018). For example, in the USA the estimated cost of the national brucellosis eradication program was around $3.5 billion between 1934 and 1997 (Sriranganathan et al. 2009). Multiple economic implications for livestock industry and public health led to efforts to control brucellosis in endemic and low-income countries through different approaches that will be discussed in the next sections (McDermott et al. 2013; Islam et al. 2018a).

## 4. *Brucella* spp. shedding in milk

The contamination of dairy products by *Brucella* species showed variable prevalence according to the studied country and geographical area (Dadar et al. 2019b). For example, brucellosis prevalence in middle and low income countries is high because of various implicated livestock species, different management systems, and specific national or regional veterinary and medical programs (McDermott et al. 2013). It should be noted that different indirect and direct methods, with various sensitivity and specificity have been applied for detection of *Brucella* species in dairy products, although the bacterial isolation is still recognised as the “gold standard” for brucellosis diagnosis. The prevalence of *Brucella* spp. in contaminated milk appeared to be of great value for risk evaluation in high risk populations considering the fact that *B. melitensis* and *B. abortus* usually infect humans through the consumption of contaminated milk products from cattle, camel, goat or sheep (Franc et al. 2018; Dadar et al. 2019b, 2020a). It has been reported that the incidence rate of *Brucella* spp. remains high in different middle income countries, including Iran (Moosazadeh et al. 2016; Alamian and Dadar 2019; Dadar et al. 2019a), Nigeria (Bale et al. 2003; Salisu et al. 2017), India (Aulakh et al. 2008; Proch et al. 2018), Turkey (Gulbaz and Kamber 2016; Kaynak-Onurdag et al. 2016), Brazil (Langoni et al. 2000; Lemos et al. 2018), Pakistan (Ali et al. 2013, 2014), Egypt (Wareth et al. 2014, 2017), China (Ning et al. 2013), and Bangladesh (Islam et al. 2019). Furthermore, *Brucella* spp. contamination was reported in milk of several animal species such as cattle, water buffalo, sheep, goats and camels. Several risk factors were identified including animal species, age, and pregnancy conditions as well as the occurrence of reproductive disorders. Furthermore, some environmental conditions, including milk storage and hygienic conditions, herd size, study area, and breeding approaches represent other key factors (Omer et al. 2000; Ning et al. 2013; Ness et al. 2017; Deka et al. 2018; Proch et al. 2018; El-Wahab et al. 2019; Dadar et al. 2019b). However, the incidence of brucellosis in different hosts and countries is directly associated with eradication and control programs in livestock that should be implemented by national veterinary services such as vaccination and “test and slaughter” policy.

## 5. Brucellosis control strategies

According to the World Health Organization (WHO), brucellosis is classified as one of the seven neglected zoonotic disease involved in a high portion of poverty in developing countries (Pérez-Sancho et al. 2015). In addition, a control program for brucellosis outbreak is valuable in preservation of dairy herd. All or some of the following control programs such as sanitation, test and removal approaches and/or vaccination can be used for brucellosis control programs (Olsen and Stoffregen 2005). Furthermore, vaccination of cattle between 4 and 12 months of age as well as cattle over the ages of 12 months is the most economic measure for brucellosis control (Nicoletti 1984). However, vaccination alone is not acceptable for the elimination of brucellosis in any host species (Olsen and Stoffregen 2005). Currently, RB51 and S19 are the live vaccine strains of *B. abortus* that more widely applied to control brucellosis in cattle (Beauvais et al. 2016; Hou et al. 2019). Moreover, the most effective strategy for eradication and control of brucellosis in young and adult small ruminant animals is with the *Brucella melitensis* REV-1 vaccine. This approach is evaluated as the most efficient in type of extensive or nomadic husbandry and in cases that brucellosis prevalence is high among small ruminants (Minas et al. 2004; Godfroid et al. 2013). Vaccine coverage and vaccine efficacy is critical to prevent Brucella infections in small ruminants and appear to be the key factor for the success of *B. melitensis* control programs (Beauvais et al. 2016). The planned control program need to evaluate numerous factors such as understanding of regional and local variations in animal epidemiological patterns of the brucellosis, cross-sectoral brucellosis epidemiological coordination and surveillance, husbandry practices, the level of infrastructure support, community awareness, and social customs (Seimenis et al. 2019). In countries showing a low prevalence of bovine brucellosis, a test-and-slaughter strategy can be applied in order to control the diseases in dairy farms (Tesfaye et al. 2011). Other preventive strategies such as the certification of brucellosis free herds and the vaccination of female bovines, have also been reported as effective approaches for brucellosis control (Renukaradhya et al. 2002; Herrera et al. 2008; Blasco and Molina-Flores 2011; Avila-Granados et al. 2019). In this respect, strict national surveillance programs are necessary to recognize infected herds, and would allow any subsequent corrective and preventive measures to be taken in (Renukaradhya et al. 2002; Rivera et al. 2002). Finally, an effective brucellosis control of animal needs different approaches such as animal surveillance by serological tests to determine infected animals, the control of brucellosis transmission to non-infected animal herds as well as the elimination of the animal carriers of the bacteria such as dog, cat and mice in the herd to eradicate the sources of infection (Gwida et al. 2010; Kiros et al. 2016). The cooperation and support of farmers are crucial for implementing long-term eradication and control programmes. Therefore, veterinary organizations should increase farmers' awareness regarding preventive strategies and transmission routes through continuous education and training programs. The accessibility to necessary resources needed for prevention and appropriate veterinary services are also important requirements.

## 6. Risk factors of *Brucella* spp. infection in dairy cattle farms

It was shown that the identification of brucellosis risk factors in livestock is of overwhelming importance to prevent the spread and persistence of this disease in different regions (Ning et al. 2013; Moosazadeh et al. 2016). A cross-sectional study including 99 dairy cattle herds (1294 female cattle sampled) was performed to define main risk factors for brucellosis infection in herds located in the suburbs of Asmara, Eritrea using a multiple betabinomial regression model (Omer et al. 2000). A variety of potential risk factors have been identified such as farm size, herd size, stocking density, type of herd, methods of disposal of manure, purchase source and frequency and the use of calving pens. In addition, mixed farming and the presence of horses or other animals (dog, sheep, cat, poultry, monkey) in the farm as well the type of service used for breeding (natural or artificial insemination), type of labor used (family or hired members) and the use of permanent housing for cows were also considered in this study (Omer et al. 2000). The authors concluded that stock density and herd type were independently related to the prevalence of *Brucella* spp, while herds with mixed-breeds were more likely to be seropositive in comparison with the herds subjected to exotic breeds (Omer et al. 2000). Another study also confirmed that mixed farming and larger herd size were the main risk factors elevating cattle Brucella infections (Al-Majali et al. 2009). It was shown in a study performed in different areas of Zimbabwe that geographical location, *Brucella* seropositivity, large herd size and frequent cattle purchase are major risk factors influencing the abortion incidence in small household herds (Matope et al. 2011a). Moreover, a total of 1,440 cattle from 203 herds were evalued in order to define risk factors associated with the presence of elevated levels of *Brucella* antibodies and showed that the application of stamping out program, promoting the use self-contained units and testing programs in livestock before movement are important to decrease the risks related to *Brucella* infections in dairy farms (Matope et al. 2011b). Pathak and his colleagues (2016) also reported that inadequate floor space and the lack of knowledge about brucellosis were important risk factors for bovine brucellosis transmission (Pathak et al. 2016). Poor implementation of critical control programs for animal brucellosis such as reporting disease to the veterinary services, testing of animals and restricting movement of infected cattle also has been reported in Cameroon as the associated risk factors of brucellosis (Awah-Ndukum et al. 2018). Furthermore, a good health system and active involvement of the populations at risk are also lacking in this region. Recent epidemiological studies showed that the sex and breed of dairy cattle, abortion history, abortion period along with farm location (farms located on steeper terrains) and farmer knowledge about the occupational risk of brucellosis had important influences on the brucellosis incidence among dairy cattle population (Halliday et al. 2015; Akinseye et al. 2016; Geresu et al. 2016; Carbonero et al. 2018). In addition, a cross sectional study around Alage district of Ethiopia revealed that the seroprevalence of bovine brucellosis and associated risk factors are significantly linked to the sex, age, reproductive status, calving interval, and number of service per conception (Asgedom et al. 2016). The survey of *Brucella* risk factors among 68 dairy farms with no positive cases of bovine brucellosis and 13 dairy farms with positive results for the Brucella Rose Bengal test (RBT) was carried out. It was shown that having a history of reactor cattle to brucellosis and sharing of water sources for cattle with and within outside farms are important risk factors for *Brucella* infection (Tukana and Gummow 2017).

Also, another study revealed that farmers did not hesitate to sell cows that experienced abortion, thereby representing a significant risk factor and neglected culprit in the spread of the disease (Asakura et al. 2018). The abortion of these cows may occur because of significance hazards; therefore, the analysis of the behaviour and perception of cattle keepers would provide important data in potential brucellosis control programs. The evaluation of brucellosis risk factors in peri-urban dairy farms of different parts of Indian cities also confirm that seroprevalence is significantly affected by the husbandry system. Increased risk could be related to intensive farming practices, often using artificial insemination methods which represent important risk factors (Lindahl et al. 2019). However, the surveillance of bovine and caprine brucellosis in most endemic countries in Africa is commonly poor. Low or poor income communities and lack of public awareness also have been mostly related to the understimation of brucellosis (Halliday et al. 2015).

## 7. Detection methods and identification of *Brucella* spp. in the milk of infected cattle, sheep, goats and camels

The detection of *Brucella* spp. in the milk and dairy herds is of overwhelming public health and economic importance (Junaidu et al. 2011; Bano and Lone 2015; Dadar et al. 2020a). Although, specialized cells of the mammary gland could synthesize milk as a sterile fluid when secreted into the alveoli of the udder (Reta et al. 2016), *Brucella* contaminations originate from within or outside the udder (Ragan and Animal and Plant Health Inspection Service 2002). An interesting study revealed the effective role of macrophages in transporting *B. abortus* from the systemic circulation into the mammary glands and milk, the intracellular multiplication in alveoli and ducts, and the transport of the organism from the infected gland to supramammary lymph nodes (Meador et al. 1989). Another possible way of milk contamination is microorganisms present in the bulk tank, on the surfaces of the milking equipment, within the farm environment as well as the interior of teats (Jayarao et al. 2004). Diagnostic methods of brucellosis are mainly according to serology tests, including SAT, RBT, CFT and iELISA as a corner stone approach, with the greatest immunological responses in different hosts for the LPS smooth chains (Nielsen 2002; Chisi et al. 2017). Serological tests are simple, inexpensive and could be rapid, although exposure to cross reacting microorganisms are susceptible to false positive reactions (Nielsen 2002; Weiner et al. 2010). The false positive reactions are known as the main diagnostic problem due to the similarity of the O-antigenic side chain of LPS of *Brucella* with other organisms such as *Escherichia coli* O:157, *Vibrio cholerae, Francisella tularensis* and *Yersinia enterocolitica* O:9. Although, serological tests such as RBT, SAT, and 2ME are often used for the first screening of brucellosis in livestock, it is now highly recommended to add complementary non-agglutination tests such as ELISA and PCR-based methods to confirm the results, which are quite more expensive. To detect *Brucella* spp. contaminations, bacterial isolation by culture is considered as the “gold standard” allowing the biotyping of the isolates (Akhtar et al. 2010; Dadar et al. 2019b). However, the culture of *Brucella* spp. needs optimal culture media conditions as well as biosafety conditions and can be challenging. Therefore, the detection of *Brucella* antibodies in milk products using *Brucella* antigens could be performed by Milk Ring Test (MRT) and immunological methods such as indirect Enzyme Linked Immunosorbent Assay (i-ELISA), that represent the conventional and most available approaches to indirectly confirm the contamination of milk with *Brucella* spp. (Altun et al. 2016; Dadar et al. 2020a). A total of 185 raw milk samples, collected from factories and dairy farms in Southwestern Uganda, were tested for *Brucella* antibodies by the i-ELISA and MRT. It revealed an equal prevalence of 27% by the two tests (Kamwine et al. 2017). However, it has been shown that serological results require more confirmation by culture and molecular approaches since presence of antibodies may not firmly indicate brucellosis infection (Karthik et al. 2014; Kamwine et al. 2017; Ali Hussein et al. 2019). PCR-based molecular techniques such as real-time PCR, PCR restriction fragment length polymorphism (RFLP) and Southern blot, Pulse-field gel electrophoresis have also proven high sensitivity in the differentiation and detection of the *Brucella* DNA (Keid et al. 2007; Brucellosis 2019; Kolo et al. 2019). The first species-specific multiplex PCR assay, named AMOS-PCR, for the differentiation of *Brucella* was designed according to the polymorphism arising from species-specific localisation of the insertion sequence IS711 in the *Brucella* chromosome (Bricker and Halling 1995). For Brucella identification, further different genes were targeted such as bcsp31(Bounaadja et al. 2009), 16S-23S rDNA Interspacer (Keid et al. 2007), recA gene (Scholz et al. 2008), and RNA Polymerase Beta Subunit (*rpoB*) (Bazrgari et al. 2020) also have been used. Another multiplex PCR assay (Bruce-ladder) also has been described for simple and rapid one-step *Brucella* identification and differentiation of most *Brucella* species and the vaccine strains *B.melitensis* Rev.1, *B.abortus* strain 19 (S19), and *B.abortus* RB51(López-Goñi et al. 2008). Moreover, real-time quantitative PCR (qPCR) appeared to be highly applicable to the analysis of the occurrence of *Brucella* contamination in milk, even allowing the discrimination of vaccination strains from virulent strains (Wareth et al. 2015; Altun et al. 2016; Kaynak-Onurdag et al. 2016).

A study performed on 250 samples of unpasteurized buffalo and cattle milk showed that iELISA and real-time PCR (RT-PCR) could effectively detect *Brucella* antibodies and *Brucella*-specific DNA, respectively (Wareth et al. 2014). The results of this study pointed to the fact that the shedding of *Brucella* spp. in milk and consumption of non-pasteurized dairy products pose an increasing risk to human consumers. Furthermore, it has been reported that the developed loop mediated isothermal amplification (LAMP) is a rapid and specific diagnostic tool for early and direct identification of *Brucella* in clinical specimens (Patra et al. 2019). In addition, several methods for the optimization of DNA extraction from milk to improve subsequent PCR analysis have been set up leading to the reliable detection of *Brucella* spp. in milk of infected animals (Leal-Klevezas et al. 1995; Ali et al. 2014; Gulbaz and Kamber 2016; Kaynak-Onurdag et al. 2016; Alamian and Dadar 2019). The sensitivity and specificity of the PCR were higher when compared to the culture method, allowing the detection of lower concentrations of *Brucella* organisms in milk (Nimri 2003; Amoroso et al. 2011; Ning et al. 2012). This sensitive and rapid approach has been used for the detection and differentiation of *Brucella* spp. in camels milk (Sprague et al. 2012; Alamian and Dadar 2019), as well as in goat, sheep, and cow milk (Hamdy and Amin 2002; Dadar et al. 2019a). Furthermore, PCR-based methods save about 3 days in *Brucella* detection compared to incubation-based methods (Kaden et al. 2018).

## 8. *Brucella*-associated public health concern in the milk supply chains

Acute or sub-acute phases of human brucellosis commonly cause an undulant fever with malaise, prostration, anorexia, sweating, and muscle pain (Manchester 1942; Shehabi et al. 1990). Specific occupations such as of veterinarians, laboratory workers, farmers, butchers, abattoir workers, and meat inspectors are at high risk of *Brucella* infections. The consumption of contaminated raw milk, cheese and butter could also transmit this disease (Dadar et al. 2020a). A significant relationship exists between the availability of economic resources and the status of brucellosis; as recently shown that the GDP per capita has a clear impact on bovine brucellosis incidence (Dadar et al. 2019b). The implementation of appropriate control policies and surveillance programs play an effective role in the reduction of the prevalence of human brucellosis (Cárdenas et al. 2019). However, *Brucella* contamination of milk products still represent an important public health issue in several areas of the world as its prevalence remains difficult to estimate (Dadar et al. 2019b). For example, informally marketed cattle milk in Uganda is a risk factor for human brucellosis, showing the importance of an information campaign related to the use of raw milk (Hoffman et al. 2016). Furthermore, application of strict surveillance and control policies of milk dairy products and milk pasteurization could decrease significantly the brucellosis incidence in humans (Mailles et al. 2016). As demonstrated in different studies, the Middle Eastern countries are highly impacted by brucellosis and contaminated milk products were responsible for several outbreaks of brucellosis in man over the last two decades (Seimenis et al. 2019). Also, a history of raw milk consumption and/or raw dairy products was reported in 63% of patients suffering from brucellosis in Turkey (Buzgan et al. 2010). Other Turkish studies estimated that the consumption of infected milk products is responsible for 62–94% of human brucellosis cases in Turkey (Gür et al. 2003; Buzgan et al. 2010). The consumption of contaminated raw milk was also responsible for 63% of human brucellosis cases in Oman (El-Amin et al. 2001), 69% in Kuwait (Mousa et al. 1988) and 57% in Iran (Moosazadeh et al. 2016). The decrease of brucellosis prevalence in livestock could be achieved by avoiding small ruminants and cattle mixing, controlling abortion rate, and culling infected animals after testing. This could lead to a significant decline in *Brucella* contaminations of milk products, thus considerably reducing human infections (Ning et al. 2013). A cross-sectional sero-survey in different villages of Punjab has reported that vaccination of household livestock could decrease the incidence of human brucellosis. Additional measures such as appropriate education related to biosecurity around abortions/calving for health-care workers as well as boiling all milk prior to consumption play an important role for the control of *Brucella* infections (Mangtani et al. 2019). Nevertheless, in urban areas with restricted contact of livestock with human populations, and lower density of animals, the appropriate education and control programs about the use of raw milk products as well as systematic pasteurization of milk products result in an effective preventive strategy to decrease the incidence of human brucellosis.

## 9. The increase of milk production in dairy farm under brucellosis control programs

Brucellosis is a reproductive zoonotic disease that is responsible for high percentage of culling (reported from 34–62%) in dairy cattle due to infertility and abortion particularly in the second lactation (Herrera et al. 2008). It was shown that the incidence of culling due to abortive disorders reached 11% among dairy cattle in Mexico (Herrera et al. 2008). It is proposed that brucellosis is the main reason of culling for abortion, although there is no available information on its etiology. Moreover, the presence of adequate veterinary services and the use of disinfectants were reported as protective factors against brucellosis in cattle of Jordan (Al-Majali et al. 2009). The most important strategy to control or eradicate brucellosis comprises preventive, test and removal programs including sanitation, vaccination as well as whole herd depopulation (Olsen and Stoffregen 2005). The current approach for brucellosis control in different ASEAN countries (Indonesia, Malaysia, Philippines, Singapore, Thailand, Brunei, Laos, Myanmar, Cambodia and Vietnam) is ‘Test-and-slaughter’ strategy. Although this approach is expensive for farmers and governments, it represents an effective protocol for eradicating all emerging and re-emerging zoonotic livestock diseases when the disease prevalence is low (not exceeding 2%) (Zamri-Saad and Kamarudin 2016). The screening test for identification of infected herd or farm is the RBT, although the complement fixation test (CFT) is often applied as a complementary confirmation test (Coelho et al. 2011; Zamri-Saad and Kamarudin 2016).

Livestock vaccination is among the effective programs used for the prevention and control of brucellosis. In endemic areas, vaccination is often used to reduce the incidence of infection and is of overwhelming importance. Cheap and effective life attenuated vaccines such as *Brucella melitensis* Rev 1 (Rev1), for sheep and goat, and *Brucella abortus* strain 19 (S19) and *B. abortus* strain RB51 (RB51) for cattle have been applied successfully in different countries (Ficht et al. 2009; Xie et al. 2018). However, vaccination alone is not sufficient for the eradication of brucellosis in livestock and should be complemented by a strict test and stamping out the program to completely eliminate the disease. The impact of vaccination on serological screen tests for brucellosis should also be taken into consideration for some vaccines such as RB51. The interpretation of false positive serological results among RB51 vaccinated cattle in endemic zones is still challenging as it is not always clear whether detected antibodies are resulting from pathogenic *Brucella* infections or passive exposure to *Brucella* antigens (Herrera et al. 2008).

A study, quantifying the production of milk in a dairy cattle population over a 6-year control program for brucellosis in Mexico, revealed that a close association exists between the increase in milk production and the application of a brucellosis control program (Herrera et al. 2008). In parallel, an increase in yearly calving was observed due to a significant decrease of abortions. Furthermore, there were lower retained placentas with healthier cows that improve the productivity of the dairy cattle. Another investigation also reported that there were no milk losses due to brucellosis in dairy Holstein herds vaccinated with both RB51 and S19 strains (Mellado et al. 2014). In vaccinated herds, it is suggested to use supplemental tests like radial immunodiffusion, beside conventional card and Rivanol tests, in order to maintain the accuracy of diagnostic serological tests (Mellado et al. 2014). Furthermore, the cattle vaccination strategy should be reevaluated in *Brucella*-endemic settings where mixed cattle flocks and small ruminant predominate (Beauvais et al. 2016). However, for the control of the spread of brucellosis infection to other localities, it is important to control the movement of animals, particularly for livestock kept under husbandry mixed system including animals of different sex, ages, pregnant and aborted population. In this context, the detection and elimination of the reservoirs, vaccination of young heifers, and regular surveillance tests to identify infected animal herds should be done to prevent the spread of the disease (Rolfe and Sykes 1987; Ragan and Animal and Plant Health Inspection Service 2002; Ocholi et al. 2005).

## 10. Failure of control of *Brucella* spp. infection in a dairy herd

The prevalence study on vaccinated herds is required to obtain a comprehensive understanding of the *Brucella* spp. circulating among livestock population (Azzam et al. 2009; Lindahl-Rajala et al. 2017). Isolation of a field strain of *B. abortus* biovar 1 from dairy cattle vaccinated with the RB51 strain showed a failure of the vaccination that could led to the re-emergence of brucellosis (Wareth et al. 2016; Alamian et al. 2020). Moreover, brucellosis control may be difficult to achieve in some developing countries without securing necessary resources and financial support (Sulima and Venkataraman 2010). Another study revealed that failure in the control of *Brucella* infections could be due to the presence of infected rats and dogs, latently infected heifers, bad hygienic conditions, and the administration of RB51 vaccine that does not confer complete protection against *B. melitensis* infection (Azzam et al. 2009). To guarantee better protection in the vaccination of small ruminants and cattle with *B. melitensis* Rev1 as well as *B. abortus* S19, a comprehensive understanding of the circulating field isolates of *Brucella* spp. among the livestock is necessary (Lindahl-Rajala et al. 2017). For this purpose, the use of a panel of screening tests is suggested as the presence of *Brucella* spp. in seronegative dairy cattle (reported by RBPT), was confirmed in milk samples of dairy cattle by bacteriological test and a PCR technique (Zowghi et al. 1990; Islam et al. 2018b). Furthermore, the significant role of cats and dogs as potential vector and asymptomatic carriers in the spread of *Brucella* infections has been reported in dairy farms (Wareth et al. 2017). It is now assumed that birds, cats and dogs may infect both livestock and humans and contaminate the environment (Wareth et al. 2017). Thus, control programs and the surveillance of brucellosis need to include brucellosis control strategies for bird, dogs, and cats which could be in close contact with dairy cattle. Also, it is important to emphasize that dairy farms using artificial insemination or natural breeding with non-certified bulls for brucellosis are subject to higher brucellosis risks (Cárdenas et al. 2019). In the absence of highly effective vaccines and because of difficulties in executing a segregation and slaughter policy of infected animals in endemic countries, control of bovine brucellosis remains a real challenge in many regions (Deka et al. 2018).

## 11. Implementing ‘One Health’ as an integrated approach to brucellosis control in endemic areas

Beside the extensive control programs from veterinary organization, it is thought that brucellosis remains a considerable issue amongst rural and small-scale livestock farmers in the world (Lindahl et al. 2014; Montiel et al. 2015). Because of the persistence of the pathogen in multiple host species, it remains widespread and neglected in different areas despite significant improvements in technology, management, and diagnostic methods over the last decades (Plumb et al. 2013). Therefore, there is a need for critical consideration on the pathogenesis, diagnosis and epidemiology of *Brucella* to develop management and prevention of brucellosis at local, national, regional and global levels (Plumb et al. 2013). It has been suggested that the core competences of an ‘One Health’ approach for brucellosis could be significantly grouped according to ecological, medical, policy and socioeconomic factors (Mazet et al. 2009). ‘One Health’ approach highlights education, science, and management factors to evaluate brucellosis by examining issues. Although, ecological parameters are not supposed to play an important role in the persistence of *Brucella* infection, whereas environmental drivers such as the seasonal variations, animal group size, behavior and host density may influence the transmission trends of brucellosis (Schumaker 2013; Dadar et al. 2020b). Determining the risks of *Brucella* spp. transmission may be deeply complicated because they are impacted by different factors such as livestock and wildlife population sizes, location, disease prevalence in wildlife, the susceptibility of livestock, and seasonal frequency of spatiotemporal interactions (Plumb et al. 2013; Schumaker 2013). Besides, reliable data on *Brucella* transmission and epidemiology dynamics in different systems, modification of brucellosis prevalence under different control options, economic evaluation of brucellosis on public health and livestock, different control strategies for improvement economic benefits in the animal and human populations could help to the cost assessment of brucellosis in livestock (McDermott et al. 2013).

Therefore, international standards to balance reporting, testing, vaccines, as well as control and prevention measures for protecting animal and human health, is an important factor in acceptable and transparent brucellosis management processes. However, specific multidisciplinary ‘One Health’ actions could provide further value regarding conventional or one-disciplinary health activities (Buttigieg et al. 2018). ‘One Health’ methods may improve decisions on resource distribution by systemic organization, collaboration, transdisciplinary communication and leadership clarity, as well as essential co-ordination on eradication program of brucellosis after several failed attempts (Mazet et al. 2009; Godfroid et al. 2013; Buttigieg et al. 2018).

## 12. Conclusions

Brucellosis is considered as one of the most important and widespread zoonotic diseases worldwide. Milk production shows a permanent increase due to the increasing demand for milk and growing population. However, the farmers’ knowledge about the zoonotic preventive practices is still limited. This review aimed at defining how brucellosis control programs could improve dairy production, in terms both of quality and of quantity.

The critical role that can play the implementation of appropriate regulatory practices to control the transmission of the disease and to reduce multiple risks factors associated with brucellosis in dairy farms has been described in detail. Considering the potential public health implication and important economic losses associated with this widespread zoonotic diseases, strict preventive programs should be performed to protect the cattle population from *Brucella* infections. According to our investigations, multiple factors influence the epidemiology of brucellosis among dairy herds, including management and trade systems, climatic conditions, and agro-ecological zones. All potential risk factors need to be carefully identified and their individual and combined impacts on milk production evaluated in order to design adequate preventive strategies and control programs to improve milk production process in endemic regions. Furthermore, governmental bodies such as veterinary organization and public health authorities should collaborate to manage brucellosis in dairy farms and to reduce the risk of human exposure. In addition, vaccine-based control programs including calf, sheep and goat vaccination appeared to be crucial in endemic areas.

An ‘One Health’ strategy including the development of veterinary capacities/services and the expansion of health education has proved remarkably effective in the control of brucellosis. Furthermore, governmental organisations and regulatory authorities should make efforts to inform farmers about the risks of the replacement of heifers and the introduction of semen from not certified farms. Thus, we recommend control programs within the ‘One Health’ principles to significantly reduce the burden of brucellosis in dairy farms through national vaccination, brucellosis testing and prevention education, while improving public health capacities and international collaborations across endemic regions.
